# Early-Life Origins of Type 2 Diabetes: Fetal Programming of the Beta-Cell Mass

**DOI:** 10.1155/2011/105076

**Published:** 2011-10-24

**Authors:** Bernard Portha, Audrey Chavey, Jamileh Movassat

**Affiliations:** Université Paris-Diderot, Sorbonne-Paris-Cité, Laboratoire B2PE (Biologie et Pathologie du Pancréas Endocrine), Unité BFA (Biologie Fonctionnelle et Adaptive), EAC 4413 Centre National de la Recherche Scientifique, 75205 Paris, France

## Abstract

A substantial body of evidence suggests that an abnormal intrauterine milieu elicited by maternal metabolic disturbances as diverse as undernutrition, placental insufficiency, diabetes or obesity, may program susceptibility in the fetus to later develop chronic degenerative diseases, such as obesity, hypertension, cardiovascular diseases and diabetes. This paper examines the developmental programming of glucose intolerance/diabetes by disturbed intrauterine metabolic condition experimentally obtained in various rodent models of maternal protein restriction, caloric restriction, overnutrition or diabetes, with a focus on the alteration of the developing beta-cell mass. In most of the cases, whatever the type of initial maternal metabolic stress, the beta-cell adaptive growth which normally occurs during gestation, does not take place in the pregnant offspring and this results in the development of gestational diabetes. Therefore gestational diabetes turns to be the ultimate insult targeting the offspring beta-cell mass and propagates diabetes risk to the next generation again. The aetiology and the transmission of spontaneous diabetes as encountered in the GK/Par rat model of type 2 diabetes, are discussed in such a perspective. This review also discusses the non-genomic mechanisms involved in the installation of the programmed effect as well as in its intergenerational transmission.

## 1. Perinatal Risk Factors for Diabetes in Later Life

Type 2 diabetes mellitus (T2D) is a complex polygenic disease that often manifests years before eventual clinical diagnosis [1]. T2D develops as a result of a failure to adequately increase beta-cell function and mass to meet the demands of prevailing insulin resistance [2]. The contribution of beta-cell failure to the pathophysiology of T2D is supported by islet pathology that reveals a beta-cell deficit of approximately 50 and 65% in individuals with impaired fasting glucose and T2D, respectively [3]. Consistent with these observations, most genes linked to T2D by genome-wide association scans have been shown to influence some aspects of beta-cell biology, such as regulation of beta-cell secretory function and development and growth of beta-cell mass [4]. It has long been recognized that nutrient availability during fetal and early postnatal life is an important determinant of adult health [5].

There are strong arguments showing that T2D is more prevalent among subjects that were in utero exposed to maternal diabetes (IUED). The role of maternal inheritance in T2D has been reported in a majority of epidemiological studies [6, 7]. To determine the role of the intrauterine diabetic environment per se, the prevalence of diabetes was compared in Pima nuclear families in which at least one sibling was born before and one after the mother was diagnosed with T2D. Offspring born after their mother displayed diabetes had a fourfold higher risk of diabetes and a higher body mass index (BMI) than their full siblings born before their mother developed diabetes [8]. These findings indicate that intrauterine exposure to a diabetic environment increases risk of obesity and T2D beyond that attributable to genetic factors, at least in Pima Indians. To circumvent the confounding effect of genes linked to early onset T2D and transmitted by the pregnant T2D mother, the effect of fetal exposure to T1D was evaluated in adult offspring lacking T1D immunological markers. A 33% prevalence of IGT was reported in offspring of T1D mothers compared with none in offspring of T1D fathers (control group) [9]. Altogether, these findings suggest that fetal exposure to maternal diabetes is indeed associated with abnormal glucose homeostasis in offspring and may participate in the excess of maternal transmission in T2D. In adult Pima Indians with normal glucose tolerance and who had been exposed to an intrauterine diabetic environment, acute insulin response to i.v. glucose was found reduced in those offspring whose mother was diabetic before pregnancy while it remained normal in those whose mother developed diabetes after pregnancy, [10]. Body fat and insulin sensitivity (euglycemic hyperinsulinemic clamp) were similar in the two groups of subjects [10]. In the same study, acute insulin response was found reduced in offspring of parents (mother or father) with early onset of T2D [10], suggesting that gene(s) linked to early-onset diabetes is(are) associated with reduced insulin secretory response to glucose [11]. Offspring of T1D mothers had reduced insulin secretion, more pronounced in IGT subjects, but similar fat mass and insulin action compared with offspring of T1D fathers [9]. Also in nondiabetic offspring of mothers with young-onset T2D (diagnosed under age 50), beta-cell function (early insulin release after oral glucose) was found decreased as compared to that of offspring of fathers with young-onset T2D [12]. Therefore, human studies suggest that insulin secretion defect participates in the abnormal glucose tolerance observed in adult offspring exposed to maternal diabetes during fetal life. Importantly, they showed that insulin secretion may be reduced even in normal glucose-tolerant offspring. Nevertheless, in children and adolescent offspring, insulin resistance involvement was suggested and may be related, at least in part, to their higher body weight.

Beside studies in IUED populations, prenatal nutrient insufficiency resulting in low birth weight is also associated with increased risk for development of obesity, cardiovascular disease, and T2D [13–15]. The association between low birth weight and development of T2D was first reported in classic studies by Hales et al. [15] that demonstrated a severalfold increase in the incidence of glucose intolerance and T2D in adult males that were born small compared with those who were born at a normal birth weight. These seminal observations since have been consistently reproduced by numerous investigators worldwide [16]. Although epidemiological evidence linking low birth weight with increased susceptibility to T2DM is strong [16], the molecular and physiological mechanisms underlying this association are still under investigation [17]. It has long been appreciated that low birth weight is associated with adult insulin resistance, which can contribute to the increased risk in development of T2D [18]. However, susceptibility to T2D in low-birth-weight individuals has also been hypothesized to be attributed to inadequate beta-cell mass formation [15]. Because it is not possible to measure beta-cell mass in vivo, this hypothesis cannot yet be tested directly in humans. However, evidence suggests that inadequate beta-cell formation in utero may underlie subsequent susceptibility for T2D. First, the fetal period is critical for endocrine pancreatic development in rodents and humans [19]. Second, clinical data show that children and adults with low birth weight demonstrate impaired beta-cell function compared with their normal birth-weight counterparts [20, 21] and human fetuses with severe growth retardation, have a reduction in pancreatic endocrine cell mass [22].

In this paper, we discuss the evidence for beta-cell dysfunction in IUED (in utero exposed to maternal diabetes), IUEO (in utero exposed to maternal overnutrition) and IUGR (in utero growth restriction) animal models, focusing on the strengths and limits of each, in order to define critical periods and types of alterations that can lead to impaired beta-cell function. We also discuss several potential mechanisms dissected in relevant animal models that begin to explain this outcome.

## 2. Compromised Intrauterine Environment and Risk for Diabetes in Later Life

Thanks to abundant studies mostly in rodents in which the foetal environment can be manipulated, a substantial body of data now addresses the mechanisms involved in the developmental programming of glucose intolerance and T2D.

**IUED Models**

In rat, maternal diabetes may be induced experimentally by streptozotocin (STZ) injection that selectively destroys beta-cells. Mild or severe diabetes ensue depending on the dose used. At birth, the progeny of mild diabetic mothers had normal weight or slight macrosomia and an enhanced percentage of pancreatic endocrine tissue due to hyperplasia and hypertrophy of the islet cells [24, 25], leading to a higher beta-cell mass that was hyper-vascularized [26]. The pancreatic insulin content and insulin secretion were raised in these fetuses [27]. On the other hand, fetuses from severe diabetic dams were small at birth and had decreased pancreatic weight [28]. Their beta-cells were almost degranulated, leading to low pancreatic insulin content and low plasma insulin [27]. Similar endocrine pancreas/beta-cell alterations with low beta-cell mass have been reported in fetuses from spontaneous diabetic BB rats [29] or spontaneous diabetic GK rats [30, 31]. The long-term consequences have been evaluated in the progeny of these models. Impaired glucose tolerance was observed in the offspring of mild STZ diabetic rats due to lower insulin secretion in response to glucose, while insulin resistance was reported in the offspring of the severe STZ diabetic mothers [32–34]. Glucose tolerance was also impaired in offspring of normal mothers receiving glucose infusion during late gestation, and it was associated with decreased glucose-induced insulin secretion [24, 35–37].

The greatest difficulty in most animal models of diabetic pregnancy has been the attainment of a stable degree of mild hyperglycemia during gestation. Though useful, most techniques used to achieve models of diabetes in pregnancy have some drawbacks. Maternal glucose infusions limited to the last trimester of pregnancy result in hyperglycemia and hyperinsulinemia and do not mimic the relative insulin deficiency of gestational diabetes [38]. The multiple lipid and protein abnormalities associated with diabetes may be as important in the induction of fetal abnormalities as hyperglycemia, but they are not replicated by the maternal glucose infusion model. A concern of studies using STZ during pregnancy is the possibility that the toxin might cross the placenta and be directly harmful to the fetal pancreas and other fetal tissues, and thus make any analysis of the long-term effects of hyperglycemia in utero difficult [39]. The problem may be circumvented by giving STZ to female neonates who will later become pregnant: this will result in moderate gestational hyperglycemia [40]. Finally it must be recognized that none of the previously mentioned models will serve directly as a model of human gestational diabetes.

**Figure 1:**
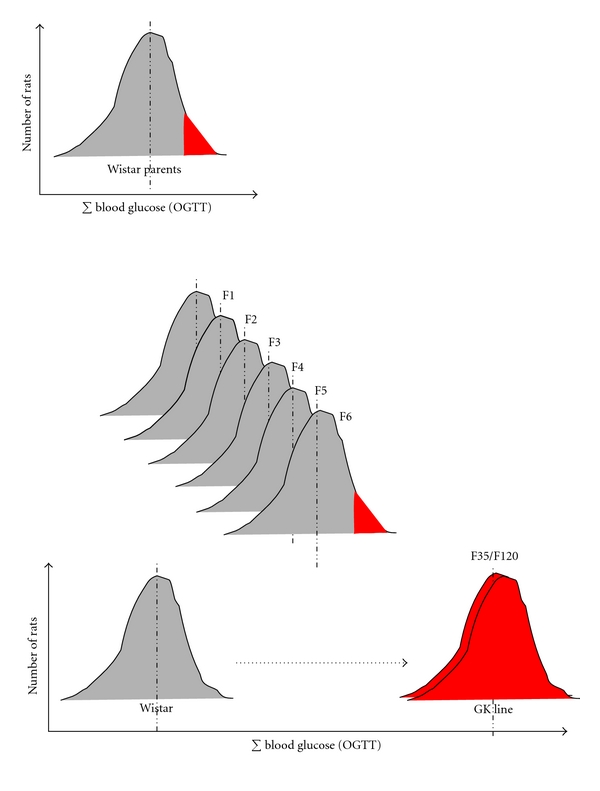
From the nondiabetic Wistar rat to the spontaneously diabetic GK (Goto-Kakizaki) rat. The inbred GK rat line (Wistar strain) was produced by Goto et al. at Tohoku University, Sendaï, Japan, by selective breeding of normal Wistar rats over many generations using glucose tolerance value (and not basal glucose value only) as a discriminant phenotype [23]. Only W rats selected at the upper limit of normal distribution for glucose tolerance were used. The diabetic state (basal hyperglycemia) was reported to become stable after the 30 generations of selective crosses in the original Japanese colony. Here is illustrated the distribution of the sum of blood glucose values (∑blood  glucose ) during standardised oral glucose tolerance tests (OGTTs) performed in original parent Wistar rats, in rats from generations F1 to F35 in the original Japanese colony and in rats from generations F35 to F120 bred under our conditions in Paris from 1989 until now (subline GK/Par). In the inbred GK/Par rat line, all rats are nonoverweight, nonketotic, and display moderate fasting hyperglycemia with strong postprandial glucose intolerance. No attenuation, nor aggravation, of the diabetic phenotype overtime (more than 20 years and 80 generations) was registered in the GK/Par line.

An ideal animal model to test the isolated impact of diabetic pregnancy would enter the pregnancy in a euglycemic state, become exposed to hyperglycaemic during whole pregnancy, and return postpartum to normoglycemic environment. Such a model also would allow study of the long-term effects of diabetes independent of any genetic influence. It was recently proposed that the pregnant GK rat being transferred normal Wistar (W) rat embryo represents a more relevant paradigm in such a perspective [41]. Using the GK/Par rat (Figure 1) we have transferred W rat oocytes to diabetic GK/Par females, and at their birth the W neonates were suckled by nondiabetic W foster mothers. Under these unique conditions, we have found that maternal diabetes negatively imprints the growth of a genetically normal (Wistar) beta-cell mass in a way as the insult is still present later at adult age as a decreased beta-cell population [42, 43].Not only maternal diabetes but also intrauterine undernutrition induced by several means such as protein (IUPR) or calorie (IUCR) restriction, or alteration in the availability of the nutrients by uterine/placental insufficiency (UPI) induced by uterine artery ligation, alter early islet development and provoke lasting consequences in rodents.

**IUCR Models**

Global restrictions (to 40–50% of normal intake) (IUCR) in the last week of rat pregnancy results in low birth weight offspring with decreased beta-cell mass. Although these animals can regain their body and pancreatic weights upon normal postnatal feeding, they still demonstrate a reduced beta-cell mass and insulin content in adulthood [44, 45]. Extending this level of nutrient restriction during suckling results in a permanent reduction of beta-cell mass [46, 47] and subsequent age-dependent loss of glucose tolerance in the offspring [48]. Underfeeding the rat mothers during the first two weeks of gestation exerts no adverse effect upon insulin secretion and insulin action in the adult male offspring [49].

**IUPR Models**

The maternal protein restriction (5–8% as compared to 20% in normal diet) (IUPR) model has been one of the most extensively studied models. The low-protein-fed mothers give birth to growth-restricted offspring [50–54], and when suckled by their mothers maintained on the same low-protein fed, they remain permanently growth restricted, despite being weaned on a normal diet [53]. Reduced placental weight and endocrine and metabolic abnormalities are also observed [50, 55, 56]. Despite young offspring of low-protein-fed dams demonstrating improved glucose tolerance [56, 57], the male offspring undergo an age-dependent loss in glucose tolerance, such that by 17 months of age they develop T2D and insulin resistance [58]. Female offspring only develop hyperinsulinemia and impaired glucose tolerance at a much later age (21 months) [54]. Studies in this model have also demonstrated reductions in beta-cell mass [51], skeletal muscle mass [53], central adipose deposit weights [57, 59], and insulin signalling defects in muscle, adipocytes, and liver [59–61]. This IUPR model has also been associated with the development of hypertension with the kidney and the rennin-angiotensin system as playing a role [62].

**UPI Models**

Fetal growth retardation may also result from experimental uteroplacental insufficiency (UPI). Fetal UPI rats have decreased levels of glucose, insulin, IGF1, amino acids, and oxygen [63–65]. UPI offspring develop diabetes in later life [66, 67] with a phenotype that is similar to that observed in T2D humans with alterations in insulin secretion and action and a failure of beta-cell function and growth [68, 69].

**IUEO Models**

There are several reports on the consequences of a high-fat diet (during gestation only or both gestation and lactation) on the adult progeny. High-fat diet consumption by female rats malprograms the male offspring for glucose intolerance and increased body weight in adulthood [70]. Some of the observed consequences include reduced whole-body insulin sensitivity, impaired or normal insulin secretion and changes in the structure of pancreas [71–74], defective mesenteric artery endothelial function [75], hypertension [76, 77], alterations in renal functions [78], increased body adiposity [72, 76], deranged blood lipid profile [71, 76, 78], hyperleptinemia [72], and proatherogenic lesions [79]. There are not many reports on fetal islet adaptations due to a high-fat dietary modification in the dam. Cerf et al. [80] demonstrated that feeding rat female with a high-fat diet throughout gestation resulted in significant decreases in beta-cell volume and number resulting in hyperglycemia in 1-day-old newborn rat pups without changes in serum insulin concentrations. However, the report of fetal hyperinsulinemia in the high-fat term rat fetus [70] is not consistent with this finding.

Maternal obesity in mice, in the absence of diabetes, can also impair glucose tolerance in genetically normal offspring. This was shown using mothers carrying the Agouti (Ay) mutation on a C57BL/6 background. On this background, the Ay mutation produces marked obesity without diabetes. At adult age while maintained on normal diet, genetically normal, adult female offspring of Ay-positive mothers exhibited reduced glucose-induced insulin secretion in vivo [81].

Also male mice whose mothers consumed a high-fat diet were heavier, glucose intolerant, and insulin resistant and produced second-generation offspring who were insulin resistant, although not obese [82]. Whether this is a consequence of paternal in utero exposure or their adult sequelae of obesity and diabetes is unclear. It was recently reported that chronic high-fat diet consumption in father rats induced increased body weight, adiposity, impaired glucose tolerance, and insulin sensitivity in their offspring [83]. Relative to controls, their female offspring had an early onset of impaired insulin secretion and glucose tolerance that worsened with time and normal adiposity. Among the differentially expressed islet genes, hypomethylation of the Il13ra2 gene was demonstrated. This is a proof of concept that paternal high-fat-diet exposure programs beta-cell dysfunction in rat F1 female offspring. This is the first report in mammals of nongenetic, intergenerational transmission of metabolic sequelae of a high-fat diet from father to offspring [83].

Among the many types of maternal metabolic stress used to produce IUGR, hypercholesterolemia combined to high fat diet was recently added since feeding LDL receptor null (LDLR^−/−^) mice with a high-fat resulted in litters with significant growth retardation. The LDLR^−/−^ high-fat diet offspring developed significantly larger atherosclerotic lesions by 90 days compared with chow diet offspring [84]. Importantly, maternal hypoaminoacidemia proved to be an important antecedent in this hypercholesterolemic IUGR mouse [84] as in a protein-deficient IUGR mouse model [84] and an IUED rat model [85]. It may be an important link in the mechanisms that contribute to adult-onset glucose intolerance, obesity, and atherosclerosis. In this study beta-cell mass was not investigated.

**Figure 2:**
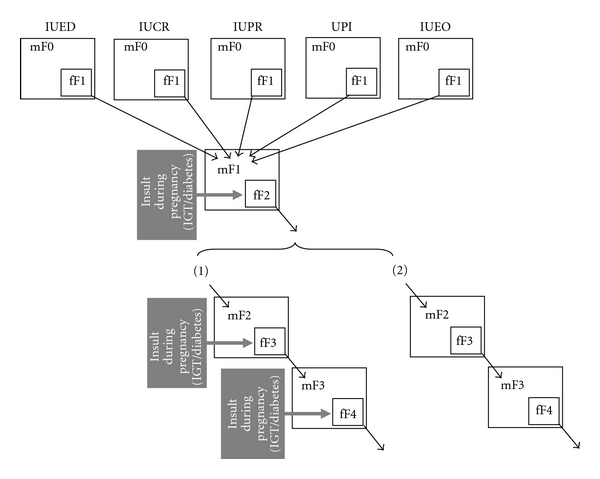
Mechanisms for the installation and intergenerational transmission of programmed beta cell mass (BCM) disruption in response to compromised intrauterine environment. The initial insults in F0 mother (mF0) impact the developing BCM of the fetuses (fF1). Diverse initial insults (IUED, IUCR, IUPR, UPI, IUEO), alone or in combination, give rise to the same programmed BCM outcome. Altered BCM phenotype in F1 females does not allow normal BCM adaptation during pregnancy and IGT/diabetes ensues (gestational diabetes). Gestational diabetes in the F1 pregnant mother (mF1) acting as an ultimate insult impacts the developing BCM of the F2 fetuses (fF2). Altered BCM phenotype in F2 females does not allow normal BCM adaptation during pregnancy and IGT/diabetes ensues (gestational diabetes). Gestational diabetes in the F2 pregnant mother (mF2) acting as an ultimate insult impacts the developing BCM of the F3 fetuses (fF3), therefore perpetuating similar BCM programming across generations. There are at least two potential scenarii for the transmission of BCM programming to subsequent generations: (1) the insult as seen in the F1 mother (IGT/diabetes insult during pregnancy) directly impairs BCM development, but BCM malprogramming is not necessarily irreversible. However, as the environmental insult (gestational diabetes) persists across generations, it recreates the same gestational phenotype in each subsequent generation (panel 1 in Figure 2); (2) the insult as seen in the F1 mother permanently affects BCM and results in the perpetuation of BCM malprogramming in the subsequent generations, in the absence of a further gestational insult (panel 2 in Figure 2). M: mother; f: fetus; F1: first-generation animals procreated by parent (F0) females submitted to experimentally disturbed metabolism during their pregnancy; F2: second-generation animals procreated by F1 females exposed to intrauterine-disturbed metabolism; IUED: in utero exposed to maternal diabetes; IUCR: in utero exposed to maternal calorie restriction; IUPR: in utero exposed to maternal protein restriction; UPI: uteroplacental insufficiency; IUEO: in utero exposed to maternal overnutrition or obesity.

To sum up, it turns to be manifest that, despite differences in the type, timing, and duration of intrauterine insult, most animal models of IUED, IUCR, IUPR, or IUEO have outcomes of impaired glucose tolerance or T2D (Figure 2).

## 3. Various Early-Life Stressors, The Same Target: The Developing Beta-Cell Mass

As abundantly illustrated in animal models, many early-life stressors such as maternal hyperglycaemia, undernutrition, overnutrition, hypercholesterolemia, corticosteroid therapy, uteroplacental insufficiency, or hypoxia trigger a beta-cell mass adaptive response in the fetus (Figure 2, Table 1).

### 3.1. Critical Windows for Adaptive Response to Early-Life Stressors

The development of the endocrine pancreas starts from a pool of common precursor cells that become progressively committed to the endocrine lineage under the control of a hierarchical network of transcription factors. During late fetal and early postnatal life, the beta-cell mass is determined by the recruitment of undifferentiated precursors, as well as the replication and apoptosis rates of the beta cells. Obviously, any disturbance of the environment of the endocrine cells at a specific developmental time-point, as it occurs in a perturbed intrauterine milieu, may modify the balance of controlling factors, thereby contributing to an adaptive beta-cell growth response which is metabolically appropriate on the short term. However, this adaptive response may turn to be detrimental if maintained on the long term, as it may foster beta-cell failure and diabetes later in life. We are largely ignorant of when programming may be initiated during development.

**Preimplantatio**

An early onset for programming was indicated, as maternal low-protein diet during only the preimplantation period of rat development (0–4 days after mating), before return to control diet for the remainder of the gestation, induced blastocyst abnormalities, and programming of postnatal growth rate and hypertension [102]. More specifically it was shown that preimplantation embryos collected from dams after 0–4 days of maternal low-protein diet displayed significantly reduced cell numbers, within the inner cell mass and trophectoderm lineages, apparently induced by a slower rate of cellular proliferation. The low-protein diet significantly reduced insulin and essential amino acid levels and increased glucose levels within maternal serum by day 4 of development. These data indicate that the mildly hyperglycemic and amino-acid-depleted maternal environment generated by undernutrition may act as an early mechanism of programming and initiate conditions of “metabolic stress,” restricting early embryonic proliferation and the generation of appropriately sized stem-cell lineages. In chemically or genetically obtained rat diabetes models in which maternal serum insulin depletion and hyperglycemia are induced, proliferation of inner cell mass or total cell numbers within blastocysts is inhibited [103, 104]. Therefore, the preimplantation embryo is particularly sensitive to metabolic modifications that may have programming consequences [105, 106], and one possibility is that it is the preimplantation embryo itself that is programmed.

**Postimplantation**

Embryo transfer experiments may also help to dissociate the impact of the maternal environment in early (preimplantation) versus late gestation (postimplantation). We recently found that embryos (blastocysts) from a nondiabetic Wistar strain placed into a diabetic GK/Par uterus develop a reduced beta-cell mass which remains low on the long term [42]. Data with rat models of prenatal undernutrition [95] also illustrate that low-energy and low-protein diets that reduce the development of the beta-cell mass in both cases act at different critical time windows. The beta-cell mass is deficient in the low-energy pancreas because this diet reduces neogenesis, probably because of high glucocorticoid levels, rather than by impairing vascularisation and proliferation. Early gestation is thus a very sensitive period in this model. By contrast, pancreatic alterations take place at a later fetal stage in the low-protein model, and the beta-cell mass is deficient in this case because this diet reduces beta-cell vascularisation and proliferation without altering beta-cell differentiation [95].

**Postnatal versus Prenata**

Further support for the crucial impact of prenatal nutritional environment is the recent report that prenatal nutrient restriction in both male and female rats led to an inappropriate postnatal beta-cell mass formation attributed to a decrease in the rate of beta-cell replication and beta-cell neogenesis [93]. In contrast, male and female rats exposed to postnatal nutrient restriction alone (with normal prenatal nutrient exposure) were characterized by decreased pancreatic and body weights, but a weight-adjusted beta-cell mass higher compared to control animals [93]. Another illustration is offered by observations in normal rat pups reared artificially on a high-carbohydrate milk formula [107]: such alteration of nutrition, during the suckling period only, induced persistent adaptation of energy metabolism in adulthood (obesity, glucose intolerance, and impaired insulin secretion).

### 3.2. Molecular Mechanisms Mediating the Perinatal Beta-Cell Adaptive Response to Early-Life Stressors

Molecular mechanisms responsible for impaired beta-cell mass formation after IUCR or IUPR have come under investigation. 

First, it has been proposed that IUCR can result in a reduction of the embryonic beta-cell progenitor pool leading to inappropriate postnatal beta-cell formation. Stanger et al. [108] demonstrated that selective genetic reduction in the size of PDX-1+ pancreatic progenitors during the fetal period results in impaired beta-cell formation during the postnatal period with consequent development of glucose intolerance during adulthood. Consistent with this, maternal food restriction leads to significant reduction in PDX-1+ and neurogenin-3+ pancreatic precursors during embryonic development in rats, diminished postnatal beta-cell formation, and inability to expand beta-cell mass in response to pregnancy [47, 94]. The UPI model is also characterized by a permanent decrease in islet PDX-1 mRNA expression. This decrease has recently been shown to be due to progressive epigenetic silencing of the Pdx1 gene locus secondary to proximal promoter methylation [69, 109], and it may be responsible for the decreased rate of beta-cell replication and inappropriate postnatal beta-cell mass development [69, 110]. In the same way of thinking, studies have demonstrated that the maintenance of methylated histone H3 Lys4 by Set7/9, a member of the SET methyltransferase family, is crucial to Pdx1 activity in beta-cell lines [111–113]. This led to the hypothesis that Set7/9 may represent a novel chromatin-modifying protein that functions in part through its recruitment to target genes by cell-specific transcription factors such as Pdx1. Since then, a role of histone methyl transferases, particularly set7, has also been demonstrated in the sustained deleterious effects of chronic hyperglycemia on human microvascular endothelial cells [114]. Such an epigenetic change could potentially be involved in the deleterious effect of high glucose upon the fetal pancreas in the IUED models.

Another mechanism proposed to explain reduced beta-cell formation after IUCR is related to prenatal glucocorticoid exposure. Administration of either dexamethasone or carbenoxolone (to inhibit 11 *β*-hydroxysteroid dehydrogenase type 2) to normal pregnant rats also causes fetal growth retardation and the adult offspring are hypertensive and hyperglycemic, with hyperactive hypothalamic-pituitary-adrenal axis [115]. Maternal undernutrition significantly increased both fetal and maternal corticosterone concentrations in rats [116]. Subsequently, maternal and/or fetal overexposure to glucocorticoids (via administration of dexamethasone) impairs both fetal and postnatal beta-cell formation in rodents and nonhuman primates [94, 117–119]. Seckl et al. [115] have shown that fetal corticosterone concentrations are inversely correlated with fetal insulin content and postnatal beta-cell formation in rats. Evidence suggests that glucocorticoids can exert a direct effect on the developing fetal pancreas via transcriptional modulation of transcription factors involved in beta-cell formation and differentiation [117]. Glucocorticoid receptors are present in the pancreas during embryonic development of rodents and humans [117], and glucocorticoids can bind to the Pdx1 promoter and thus suppress fetal endocrine cell differentiation [117]. Glucocorticoid treatment has been shown to significantly reduce fetal expression of key endocrine transcription factors such as Pdx1 and Pax6 but simultaneously increase expression of transcription factors that regulate development of the exocrine pancreas [119].

It has also been demonstrated that the UPI or the low-protein IUPR offspring experience increased oxidative stress and impaired mitochondrial function [96, 120]. The mitochondrial dysfunction was not limited to just the beta cell, as mitochondria from both the liver and skeletal muscle exhibit decreased oxidation of pyruvate, subsequently leading to the development of features commonly found in T2D [100, 121]. Also exposure to a Western-style diet before and during pregnancy (an IUEO model) alters the redox state as early as preimplantation development, leading to mild oxidative stress associated with inflammation. The finding that administration of antioxidants to the dam reverses oxidative stress and completely prevents the development of glucose intolerance and increased adiposity in the adult offspring suggests that oxidative stress plays an important role in the development of adiposity in this case [122]. Some studies in the low-protein IUPR model have demonstrated that oxidative stress is not limited to just mitochondrial DNA damage, but also to genomic DNA, impacting cell-cycle regulation and gene expression [123]. While DNA is being targeted throughout by ROS, there are particular regions that are known to be more sensitive to ROS-mediated damage, for example, telomeres. Telomeres comprise GC-rich repeats and are found at the ends of each chromosome. They are known to shorten with each cellular division and, hence, can act as a mitotic clock, registering the number of replicative divisions to have taken place within the cell. Investigations using an IUPR model have indeed reported a decrease in longevity in the offspring [123, 124] accompanied by reduction in mitochondrial antioxidant defences [96, 125] and telomere length in islets [125].

Pancreatic islet development has been shown to be influenced by a number of growth factors including the insulin-like growth factors, IGF-I and IGF-II whose expression in utero is regulated by nutrient and hormone concentrations. IUPR modifies expression of both IGF genes in a variety of fetal tissues. In an IUPR rat model with a decreased beta-cell mass and beta-cell replication and an increased rate of beta-cell apoptosis, gene expression for IGF-II but not IGF-I was found reduced in the fetal pancreas [126]. In a different IUPR model with more severe global food restriction which induced hyperinsulinemia and an increase in beta-cell mass in their fetuses [90], the fetal phenotype was unexpectedly associated with an increase in pancreatic IGF-I expression, islet IGF-1R [91], and IRS-2 [92]. In the fetal GK/Par rat exposed to mild hyperglycemia during gestation (a model of IUED), data from our group suggest that the beta-cell deficit (reduced by more than 50%) starts as early as fetal age E16 and reflects decreased beta-cell proliferation, a limitation of beta-cell neogenesis from precursors, and increased apoptosis of both beta cells and their precursors [86]. Notably, Pdx1 and Neurogenin3 expression were decreased on E18 but normally expressed on E13 [86]. Defective signalling through the Igf2/Igf1-R pathway may represent the primary instrumental anomaly since Igf2 and Igf1-R protein expressions are already decreased within the GK/Par pancreatic rudiment at E13, at a time when beta-cell mass (first wave of beta-cell expansion) is in fact normal [31]. Low levels of pancreatic Igf2 associated with beta-cell mass deficiency are maintained thereafter within the fetal pancreas [87]. Crossbreeding protocols between nondiabetic W and diabetic GK rats showed that, in late gestation (E18), pancreatic Igf2 protein expression was as low in GKmother/GKfather and Wmother/GKfather crosses as in GKmother/GKfather crosses [87]. These findings rather support the hypothesis that the pancreatic Igf2 anomaly in the GK diabetic model is linked to a genetic determinism. This view is also consistent with the results of genetic analyses that linked a locus containing the gene encoding Igf2 to diabetes in the GK rat [127]. The Igf2 gene is subjected to paternal genomic imprinting. However, because the Igf2 expression is similarly affected in fetuses, regardless of whether the father is W or GK [87], we cannot conclude with a simple change of Igf2 gene imprinting in the GK rat.

Finally, our understanding of the underlying mechanisms for reduced BCM in response to inappropriate perinatal nutrition is growing rapidly. However, the relative contribution of the many intrinsic and extrinsic factors which contribute to the adaptive response of the developing endocrine pancreas is still to be established.

## 4. Various Early-Life Stressors: One Ultimate Programming Inducer—Perinatal Hyperglycemia

As abundantly illustrated in animal models, early-life stressors such as maternal undernutrition, overnutrition, hypercholesterolemia, corticosteroid therapy, uteroplacental insufficiency, or hypoxia program metabolic adaptations that initially favour survival but are ultimately detrimental to adult health. Interestingly, there exists in fact one crucial commonality between these models with quite different etiologies: in most of the cases, the altered maternal/fetal metabolism appears to be associated with a diabetogenic effect in the adult offspring either male or female, resulting in a permanent deficiency of the endocrine pancreatic function (F1). In females, the combination of a latent diabetogenic tendency (low insulin response) and the metabolic stress of pregnancy promotes gestational diabetes. F1 gestational diabetes per se is an inducing factor for impaired glucose tolerance and gestational diabetes again in the next female generation (F2). 

Finally, the relevant message is that programming of the endocrine pancreas ultimately originates from hyperglycemia experienced during the fetal and/or early postnatal life, whatever the etiology of maternal hyperglycemia, primary (in F0 diabetic mothers) or secondary (in F1 diabetic mothers issued from F0 mothers exposed to undernutrition, UPI, or high glucocorticoid) (Figure 2).

## 5. Transgenerational Inheritance of Beta-Cell Mass Programming

While a large number of animal studies have shown the effects of undernutrition during foetal/perinatal development on the glucose metabolism of offspring (F1) in adulthood, several studies have shown that glucose metabolism is also altered in the offspring (F2) as well as grand offspring (F3) of fetally malnourished F1 females, even when the F1 and F2 females have been well nourished since weaning [32, 128] (Figure 1, Table 1). With an aim to dissect the relative parental contributions that lead to F2 offspring outcomes in these models of maternal (F0) undernutrition, it was recently reported that F1 males exhibit moderate hyperglycemia and IGT with aging and impaired glucose-stimulated insulin secretion and that all F2 offspring of F1 males or F1 females develop glucose intolerance [99]. Therefore, intergenerational progression of glucose intolerance can derive from both the maternal and paternal lines. This is an experimental proof that transgenerational transmission of IGT may also occur through the paternal lineage, beside the more widely accepted maternal and grandmaternal inheritance of diabetes [94, 99, 128, 129].

Conceptually, transgenerational inheritance of disease risk may be mediated by nongenomic mechanisms, including either (1) epigenetic mechanisms [130–133] or (2) other broader indirect mechanisms associated with parental physiology [134]. First, alterations in nutrition during development can alter epigenetic marks, thus regulating gene expression through DNA methylation and/or histone modifications. Interestingly, such epigenetic modifications may progress with aging during postnatal life, in association with metabolic phenotypes, as recently observed at the Pdx1 and GLUT4 loci in UPI rats [109, 135]. If these epigenetic changes occur in the germ line, they can be inherited through meiosis [136], thus providing a plausible explanation for intergenerational effects, transmitted via either maternal or paternal lines. In addition, other indirect biological processes may influence phenotypes in subsequent generations. For example, physical constraints may alter birth size through the maternal lineage: since uterine size is reduced in girls that are born small and remain short, this may influence fetal growth and reduce weight in their progeny [134]. 

**Figure 3:**
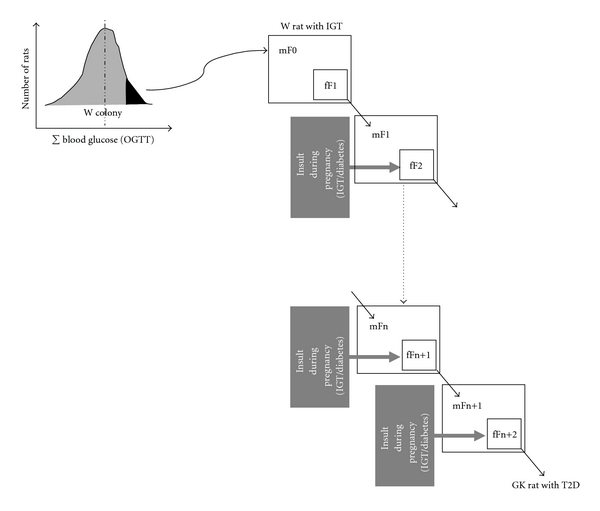
Mechanisms for the installation and intergenerational transmission of programmed beta-cell mass (BCM) disruption in the GK/Par rat model of type 2 diabetes. Maternal IGT/diabetes during gestation induces BCM programming in the first (F1) and the subsequent rat generations. Metabolic modifications in the pups during the in utero and suckling periods are followed by the onset of pathological conditions in adulthood (glucose intolerance and type 2 diabetes) and the transmission of programmed endocrine/metabolic capacities to the next generation. W: Wistar strain.

Furthermore, maternal metabolism may also influence cross-generational phenotypes [32]. Maternal undernutrition during pregnancy (F0) increases risk for developing diabetes and obesity in her offspring (F1). When these high-risk adult F1 females become pregnant, the metabolic stress of pregnancy may result in hyperglycemia and/or overt gestational diabetes that may, in turn, contribute to defective beta-cell mass and increased diabetes risk in F2 offspring [32]. By this mechanism gestational diabetes may pass from one generation to the next one. In these last examples, intergenerational transmission of phenotypes would occur exclusively through the maternal lineage, as opposed to the epigenetic mechanisms mentioned above. Such a scenario is relevant to the GK/Par rat (Figure 3), since the GK/Par mothers are mildly hyperglycemic through their gestation and during the suckling period. It offers a rationale to elucidate several clues: (1) the initiation of pancreas programming in the F1 offspring of the first founders (F0), since the GK line is issued from intercrosses between Wistar females and males with borderline IGT but otherwise normal basal blood glucose level [23]; (2) the progression of the IGT phenotype until a stable mild diabetic phenotype was reached among the generations *n* = 30 [23]; (3) the lack of attenuation of the diabetic GK phenotype overtime (along more than 20 years and 80 generations), since offspring of GK female/W male crosses were more hyperglycemic than those of W female/GK male crosses [89].

## 6. Epigenetic Mechanisms Mediating the Diabetes Risk Associated with Beta-Cell Mass Programming

Several lines of evidence indicate that epigenetic modification may be a key unifying mechanism mediating risk associated with a perturbed intrauterine environment. First, disruption of physiologic responses and functional capacity as observed in multiple tissues of IUED or IUGR animals and humans, including muscle, adipose, pancreas, liver, and CNS may be related to histone modification and DNA methylation, thereby altering related gene expression [133]. 

The preimplantation embryo is particularly sensitive to epigenetic modifications that might permanently alter the phenotype in the adult [105, 137]. For example, in the agouti mouse model, folate supplementation of the maternal diet at conception increases DNA methylation of the agouti gene and increases longevity of the offspring [138]. Maternal protein restriction has been shown to alter the methylation status of the promoters of the glucocorticoid receptor [97], PPAR*α* [98], and the angiotensin receptor [139] with parallel changes in gene expression. More recent studies have shown that histone modifications can also be influenced by the early environment. Alterations in histone modifications have also been implicated in mediating the effect of caloric restriction during the second half of pregnancy on the programmed reduction of GLUT4 expression in the offspring [135]. In the case of the UPI rat model and the pancreatic tissue, Stoffers and colleagues have reported a progressive reduction in expression of Pdx1, a key transcription factor regulating pancreatic development and function [69]. Pdx1 expression is reduced by 50% in UPI fetuses and by 80% in adult UPI offspring. Notably, these changes precede the onset of beta-cell dysfunction, suggesting a primary pathogenic role. Since the Pdx1 promoter is a target for epigenetic modification, as it contains a conserved CpG islands and is associated with high levels of histone acetylation. Interestingly, binding of both acetylated histone H3/H4 and the transcription factor USF1 was found abolished in UPI fetuses [109]. While there was methylation at multiple CpGs in UPI adult offspring, no methylation was detected in UPI neonates, indicating that methylation was unlikely to explain Pdx1 repression early in life. Together, these data indicate that progressive silencing of gene expression is largely initiated by early epigenetic changes and is maintained thereafter even in the absence of further experimental insults during postnatal life. UPI also increases histone acetylation of the PPAR*γ* coactivator PGC-1 and carnitine-palmitoyltransferase I (CPT1) promoters in newborn and young rats, and these changes are associated with increased PGC-1 and CPT1 mRNAs [101]. Finally, there is now little doubt that epigenetic regulation of gene expression also occurs in humans as a response to early nutritional insult: a recent study has revealed that individuals who were exposed to famine in utero during the Dutch Hunger Winter had altered methylation of the Igf2 gene in white blood cells in adulthood [140].

## 7. Implications for Public Health

Although the focus of most studies in the metabolic programming field has been on delineating the effects of reduced maternal nutrition, there is now a growing interest in the role of maternal overnutrition in the programming of diabetes risk. The worldwide prevalence of obesity continues to increase, in association with an increase in the risk of metabolic T2D. Indeed, a recent study estimated that the number of people worldwide with diabetes would increase from 171 million in 2000 to 366 million by 2030 if the prevalence of obesity remained constant [141], which has major implications for public health strategies worldwide [142]. This global trend to increasing obesity is reflected in the increasing numbers of women who are obese during pregnancy [143]. Given that the offspring of obese mothers have an increased risk of developing obesity and T2D themselves [144, 145], the potential impact of the intergenerational consequences of maternal obesity is of great concern for public health policy makers. 

Moreover, maternal hyperglycemia per se increases the probability of adolescent obesity and future T2D. To what extent maternal hyperglycemia is fuelling the global rise in obesity and T2D is unknown, but its contribution is highly significant. The exact degree of hyperglycemia that has this effect and the exact timing in pregnancy that hyperglycemia is impressionable on fetal programming is unknown. The need to identify and treat all women with gestational diabetes is very much dependent on us knowing this. Meanwhile, achieving rigorous glycemic control in women with diabetic pregnancy has to remain a major therapeutic goal.

Several interventions (dietary or pharmacological) to reduce the long-term sequelae of early-life programming effects have been used in animal models. For example, the administration of folic acid with a low-protein diet during pregnancy prevents the altered phenotype and epigenotype in rat offspring [97], and administration of a diet rich in methyl donors prevents the transgenerational increase in obesity in agouti yellow mice [146]. Importantly, the timing of such interventions can be crucial. Examples include neonatal leptin treatment which reverses the programming effects of prenatal undernutrition [147]. In the UPI rat model, epigenetic silencing of the Pdx1 gene can be reversed during a critical developmental window in the neonatal period, using trichostatin A which inhibit HDACs [109]. In the same model, exposure to exendin-4 in the neonatal period reversed the detrimental fetal programming of the beta-cell mass and prevented the development of diabetes in adulthood: this was closely related to restoration of pdx1 expression and beta-cell proliferation rate [69]. A GLP-1 or exendin-4 treatment limited to the neonatal prediabetic period was also shown to delay the installation and limit the severity of T2D in the GK/Par model [88]. In such context, it is important to note that GLP1-derived drugs that are currently used to treat patients with T2D may target chromatin remodelling. Treating beta cells from the INS1 cell line or dispersed mouse islet cells with GLP-1 increased global acetylation of histone H3 and increased its phosphorylation in a concentration-dependent manner [148]. Such histone modifications increased association with the transcription factor phospho-CREB and with cAMP-response CREB coactivator 2. Taken as a whole, these data may provoke optimism—that there may be a window for potential postnatal therapeutic interventions to prevent/modify the “programmed” diabetes risk.

## Figures and Tables

**Table 1:** Beta-cell (BC) mass characteristics in rodent models of compromised intrauterine environment.

Rodent models	BC phenotype		
	fetal	neonatal/suckling	adult
IUED, mild STZD (F1)	Increased BC mass, high BC proliferation [24–27]	Increased BC mass, high BC proliferation, high islet vascularisation [24, 25]	Normal BC mass, low GSIS, low GT [24]
F2 issued from mild STZD F1	NR	NR	Low GSIS, low GT [26, 32]
IUED, severe STZD (F1)	low BC mass [27, 28]	NR	Increased BC mass, high GSIS, low GT [32–34, 39]
IUED, GI (F1)	Slightly increased BC mass, high BC proliferation [35]	NR	Low GSIS, low GT [24, 35–38]
F2 issued from GI F1	NR	NR	Low GSIS, low GT [35]
IUED, GK/Par	Low BC mass, low BC neogenesis [30, 31, 42, 86, 87]	Low BC mass, low BC neogenesis [88]	Low BC mass, low BC proliferation, low GSIS [23, 88, 89]
Severe IUCR (F1)	Increased BC mass, high BC neogenesis, high BC proliferation [46, 90–92]	NR	Low BC mass, low BC proliferation, low GSIS [46]
IUCR (F1)	Low BC mass, low BC neogenesis [44, 45, 93]	Low BC mass [47]	Low BC mass, low GSIS, low GT [44–49]
F2 issued from IUCR F1	Low BC mass, low BC neogenesis [94]	NR	Low GSIS, low GT [32]
IUPR (F1)	Low BC mass, low BC proliferation, low islet vascularization [50, 51, 93, 95]	Low BC mass [93]	Low BC mass, low GSIS, low GT [51, 53, 54, 56–62, 96–98]
F2 issued from IUPR F1	Low BC mass [26]	NR	Normal GT [99]
UPI (F1)	Normal BC mass, low BC proliferation, low islet vascularization [63–65]	Normal BC mass [65–67]	Low BC mass, low GSIS, low GT [66, 67, 100, 101]
F2 issued from UPI F1	NR	NR	Low BC mass, low BC proliferation, low GSIS, low GT [32]
IUEO (F1)	NR	Slightly reduced BC mass [80]	Normal or decreased GSIS, low GT [70–76, 81–84]
F2 issued from IUEO F1	NR	NR	Normal GT [82]

NR: not reported in the literature to the author's knowledge; GSIS: glucose-stimulated insulin secretion; GT: glucose tolerance; STZD: diabetes obtained after streptozotocin administration to adult females several days before mating or during pregnancy; GI: continuous glucose infusion in unrestrained normal pregnant rat during the last week of pregnancy; F1: first-generation animals procreated by parent (F0) females submitted to experimentally disturbed metabolism during their pregnancy; F2: second-generation animals procreated by F1 females exposed to intrauterine disturbed metabolism; IUED: in utero exposed to maternal diabetes; IUCR: in utero exposed to maternal calorie restriction; IUPR: in utero exposed to maternal protein restriction; UPI: uteroplacental insufficiency; IUEO: in utero exposed to maternal overnutrition or obesity.

## Abbreviations

m: Mother
f: Fetus
F1: First-generation animals procreated by parent (F0) females submitted to experimentally disturbed metabolism during their pregnancy
F2: Second-generation animals procreated by F1 females exposed to intrauterine-disturbed metabolism
IUED: In utero exposed to maternal diabetes
IUCR: In utero exposed to maternal calorie restriction
IUPR: In utero exposed to maternal protein restriction
UPI: Uteroplacental insufficiency
IUEO: In utero exposed to maternal overnutrition or obesity
BCM: Beta-cell mass.
